# Evaluation of the probable synergistic toxicity of selected potentiated antiretroviral and antibiotics on some aquatic biomarker organisms

**DOI:** 10.1007/s10661-023-12068-x

**Published:** 2023-11-17

**Authors:** Elizabeth Oyinkansola Omotola, Bettina Genthe, Luyanda Ndlela, Olatunde Stephen Olatunji

**Affiliations:** 1 School of Chemistry and Physics, University of KwaZulu-Natal, Durban, 4000 South Africa; 2 Department of Chemical Sciences, Tai Solarin University of Education, Ogun State, PMB 2118, Ijebu Ode, Nigeria; 3 Department of Microbiology, Stellenbosch University, Stellenbosch, 7600 South Africa; 4 Natural Resources and the Environment Division, Council for Scientific and Industrial Research, Stellenbosch, 7599 South Africa

**Keywords:** Bioassays, *Allium cepa*, Antiretroviral, Mutagenicity, Chromosomal aberration, Root-tip growth

## Abstract

Environmental effects of active pharmaceutical compounds (APCs) in the environment are not well characterized, hence the need for comprehensive evaluation. This study employed three bioassays using three organisms, namely, *Allium cepa*, *Daphnia magna*, and *Salmonella typhimurium*, in the ecotoxicity study of lone and a mixture of selected APCs, namely, lamivudine (L), an antiretroviral, and ciprofloxacin (C) and sulfamethoxazole (S), antibiotics, at a concentration range between 10 and 100 ppb, in order to evaluate the potential of the lone and ternary mixture to exert synergistic toxicity. Study results from exposure to lone APCs showed that the L, C, and S trio individually had fatal impacts on daphnids, with mortality rates of 100, 75, and 95%, respectively, after 48 h. Sulfamethoxazole showed a mutagenic tendency, with a mutation ratio (background/sample ratio) of 2.0. Lamivudine showed a lethal impact on the root length of *A. cepa* (*p* > 0.05, *p* = 3.60E–3). Further microscopic examination of the *A. cepa* root tip revealed chromosomal aberrations on exposure to each compound. The LCS-mix ecotoxicology bioassays indicated a synergistic effect on the daphnids, probably due to potentiation. Although the LCS mix had a cytotoxic effect (evidenced by the absence of bacteria colonies) on exposed TA 98 P450 *Salmonella typhimurium* strain, this effect was not observed in other bacterial strains. Microscopic examination of *A. cepa* exposed to the LCS-mix revealed an aberration in the mitotic stage of the cell. The impact of combination of the pharmaceuticals in aqueous ecosystems was greater than when exposed to the tested individual pharmaceutical compounds. Study result showed that these compounds have tendencies to pose a higher risk to exposed living entities when in combined/potentiated forms, and this could lead to distortion of the regular functioning of the ecosystem, particularly bacterial and other microbial populations that are listed among primary producers of the aquatic food web.

**Supplementary Information:**

The online version contains supplementary material available at 10.1007/s10661-023-12068-x.

## Introduction

Pharmaceutical compound residues and their metabolites are released into the environment in a non-isolated state. Hence, they do not exist as stand-alone in the numerous environmental matrices they end up in. For instance, in a study conducted by Kairigo et al. (2020), about 27 APC residues were reported in a single river in the UK. The study envisaged and hypothesized that combinations of residues of these compounds in different permutations might exert much more adverse effects than they can do in their isolated (lone) states or metabolic forms due to the possible attributable chemistry (interaction) that can occur between the drugs’ residue in the environment. Such chemistry may include synergistic, additive, or antagonistic interactions resulting from chemicals which may or may not belong to the same pharmacological class but have different R-group substituents or functional moiety (compounds having the same mode of action but different properties) (Wilde et al., 2017; González-Pleiter et al., 2013. Residues of APCs from the same or different sources are generally likely to interact with each other, resulting in more complex combinations once pharmaceutical compounds are released into the environment (Wilde et al., 2017). The scale and complexity of combined/potentiated APCs in the environment are hard to establish due to the strain of specificity and the difficulty of selecting representative endpoints. Consequently, the quantitative assessment of multiple APC stressors is not straightforward, unlike a single stressor (lone PC). Thus, the contamination of the environment with residues of APCs in their lone or combined/potentiated forms, as well as with their metabolites, is concerning.

Residues of active pharmaceutical drugs and their metabolites find their way into the environment through various routes, from post-consumption elimination via sweat, urine, and feces to indiscriminate disposal of unused/expired drugs, with the significant entry route being inefficient sewage treatment plants (Pavlović et al., 2007; Aydin et al., 2019; Valdés et al., 2014; Couto et al., 2019). The frequency of detection of some active compounds of these pharmaceutical drugs (PDs) in many urban and peri-urban surface waters is well established (Agunbiade & Moodley, 2014; Madikizela et al., 2017, 2020; Matongo et al., 2015; Ojemaye & Petrik, 2019). Aside from a few reports on the presence of antiretroviral drug, lamivudine, and the antibiotics, ciprofloxacin and sulfamethoxazole, in South African environment and worldwide (Abafe et al., 2018; Omotola & Olatunji, 2020; Swanepoel et al., 2015; Wood et al., 2015), there is a high usage volume of different types of antiretrovirals and antibiotics drugs, as well as other types of pharmaceutical drug classes such as analgesics, antihistamines, non-steroidal anti-inflammatory drugs (NSAIDs), and many others. While the sources of APCs in the environment are largely known, their magnitude and impact on environmental resources are not very clear. This is because studies on the ecotoxicities of these contaminant compounds at their detected environmental occurrence levels (concentrations) are limited, hence the scarcity of ecotoxicology data and information. Also, there is a need for long-time monitoring of the condition of environmental resources and the various potential stressors influencing those resources.

This study, therefore, investigated the ecological toxicities of lamivudine, belonging to the antiretroviral therapeutic drugs group, and ciprofloxacin and sulfamethoxazole, both belonging to the antibiotics group sub-classes, fluoroquinolone and sulfonamide, respectively, in their stand-alone form and in different potentiated forms, using the *Allium cepa* (*A. cepa*), *Daphnia magna* (*D. magna*), and *Salmonella typhimurium* (*S. typhi.*) bioassays. This was carried out at a concentration range between 10 and 100 ppb. The range of concentrations reported in the authors’ earlier work was taken into consideration when choosing these concentrations (Omotola & Olatunji, 2020). These quantities were analytically verified on the liquid chromatograph-mass spectrometer during environmental monitoring of pharmaceutical compounds in the KwaZulu-Natal Province of South Africa in the previous work by Omotola and Olatunji (2020). The choice of the ecotoxicology bioassays used in these study targets the bioindicator organisms’ sensitivities to small changes in water quality, as in the case of *Daphnia* (Jeong & Simpson, 2019); *Salmonella typhimurium* (in the Ames test), a good indicator for chemicals’ mutagenicity (Ames et al., 1975); and *A. cepa* for investigating exertion of chromosomal aberrations and the capacity of the chemical to impose genotoxicity on living entities (Wijeyaratne and Wadasinghe, 2019).

## Materials and methods

### Materials

Ciprofloxacin and sulfamethoxazole standards were purchased from Sigma Aldrich, Germany, while lamivudine was procured from DLD Scientific, J and H Chemical Co Ltd., South Africa. HPLC- and analytical-grade chemicals and solvents, including methanol (MeOH), ethanol (etOH), agar–agar, glucose monohydrate, NaCl, MgSO_4_·7H_2_O, citric acid·H_2_O, K_2_HPO_4_, NaNH_4_HPO_4_·4H_2_O, l-histidine, and d-biotin, were procured from Sigma Aldrich, South Africa. Daphtoxkit (kit number DM576) was purchased from Tox Solutions Kits and Services (Stellenbosch, South Africa). *A. cepa* was purchased from South African stores, Stellenbosch. *Salmonella typhimurium* bacterial strains (TA100, TA100 P450, TA98 P450) were provided by the Council for Scientific and Industrial Research (CSIR), South Africa. Well microtiter (96) plates (Cat. No. 95029780, Thermo Fisher Scientific, Denmark) were also provided by the CSIR, Stellenbosch.

## Sample preparation

A 1000-ppm stock solution of lamivudine and sulfamethoxazole was prepared in HPLC-grade MeOH. Ciprofloxacin was not directly soluble in methanol; hence, 10 mg of the compounds was initially dissolved in 3.5 mL water and 0.1% formic acid, respectively, before dissolution in MeOH to achieve a 1000-ppm stock solution. The stock solutions were stored at –20 °C in a low-temperature freezer. Sulfamethoxazole, ciprofloxacin, and lamivudine solutions (10 ppm) were prepared in methanol from where subsequent dilutions were made to achieve appropriate relevant concentrations in simulated freshwater (for daphnids’ test), tap water (for *A. cepa* exposure), and Millipore water (for the Ames mutagenicity test). A mixture of all three pharmaceutical chemicals was also prepared by combining equal volumes of each chemical.

## *Allium cepa* root length and chromosomal abnormality assay (onion root)

The onion root length and chromosomal abnormality assays were conducted according to the method described by Barbério (2013) with slight modifications. Whole-onions test specimens were grown at room temperature (in triplicate). The onions were initially grown for 2 days in tap water. Prior to growing them in tap water, the ring of the root, the primordia at the bottom of the onion bulb, was scraped with a sterile surgical blade to remove all existing root growth. After 2 days of tap water exposure, the lengths of the root fibers of each onion were measured and recorded. This was followed by exposure to the test solutions for another 2 days (tap water was used as the control). Under controlled conditions, it is expected that the root length should double. Hence, the measured values of the most extended root length before and after exposure to test solutions were used as a criterion to evaluate if there were any adverse effects on the growth of the onion roots. At the end of the exposure, the onion roots were cut 3 cm from the tip and placed in Carnoy’s fixative solution (3 parts glacial acetic acid to one-part absolute etOH (Merck, Germany)) until further microscopic analysis.

## Microscopic examination

The fixed onion roots were washed in distilled water and hydrolyzed in 1 M hydrochloric acid (HCl), placed in a water bath set at 60 °C for 7.5 min, to dissolve substances that unite the cells, usually pectin (Nefic et al., 2013). This was followed by washing the root tips in distilled water, after which they were stained using Feulgen stain for 2–3 min. The stained root tips were then rinsed in distilled water, transferred onto clean glass microscope slides, and counter-stained with acetocarmine. The stained root tips were initially crushed with the flat end of the surgical blade (Nefic et al., 2013) and then under a coverslip, applying pressure with care to avoid movement of the coverslip or breaking the coverslip. The squashed onion root tips were thereafter imaged using a LEICA DM-750 light microscope at × 400 and × 1000 magnifications. A qualitative microscopic analysis was carried out by observing the abnormalities of the onion chromosomes, as reported by Çavuşoğlu et al. (2017), and comparing them to control chromosomes.

## *Daphnia* 24–48-h toxicity test

Acute immobilization test with *Daphnia magna* (freshwater fleas) was carried out according to the Organization for Economic Cooperation and Development (OECD) Tests No. 202 guidelines for the testing of chemicals (O.E.C.D., 2004), which forms the basis of the kits’ manufacturer’s protocol, with slight modification. The underlying principle of this test is based on whether the test solutions are toxic to the daphnids, signified by motility and mortality rates.

Freshwater was simulated using standard concentrated salt solutions (batch number ISOD070319). Sodium hydrogen carbonate (NaHCO_3_), calcium chloride dihydrate (CaCl_2_·2H_2_O), magnesium sulfate heptahydrate (MgSO_4_·7H_2_O), and potassium chloride (KCl) concentrated solutions were used to simulate the freshwater medium for the hatching of the daphnids’ and for exposure study.

## Relationship between freshwater and ephippia hatching

The ephippia were hatched within 72-h period at 28 °C, under constant illumination with the aid of cool white light. After the 72-h hatching period, the daphnids were fed with dry spirulina (algae) for a period of 2 h, after which they were harvested into a 30-well plate containing the test solutions. The test solutions were prepared in freshwater from a 1000 µg/mL stock solution of lamivudine, ciprofloxacin, and sulfamethoxazole, using serial dilutions. Freshwater containing the same amount of MeOH used in the preparation of the test solutions was used as the control. The freshwater and control water used for exposure studies were aerated with the aid of an aquarium air pump before daphnid exposure. Four replicate analyses were conducted at each test solution level, for which five active daphnids were placed in each well, and exposed over a period of 24 and 48 h. Data obtained were classified using fuzzy logic (Novák et al., 2012), allowing for consistent results interpretation (Table 1).

**Table 1:** Classification of lethal impact of targeted compounds using the fuzzy logic principle

No lethal impact ($w$)	Mild lethal impact ($x$)	Severe lethal impact ($y$)	Extremely severe lethal impact ($z$)
*m*<25	*m*≥25≤50	*m*>50≤75	*m*>75

Where $$m\mathrm{ is \% mortality of daphnids}$$

## Ames mutagenicity test

The Ames mutagenicity test detects mutations that reverse mutate test strains (*Salmonella typhimurium*) and restore the functional capability of the bacteria to synthesize an essential amino acid required by the parent test strain (Ames et al., 1975). The revertant bacteria are detected by their ability to grow in the absence of the amino acid required by the parent test strain. The Ames test method was employed to investigate the potential of a chemical to induce a mutagenic effect in exposed organisms. Three different *Salmonella typhimurium* bacteria strains ([TA98 P450], [TA100], and [TA100—P450], which simulate metabolic activation) were used in order to account for discrepancies in the way similar organisms (bacteria) may respond to test solutions.

*Salmonella typhimurium* bacteria were cultured on a freshly prepared nutrient broth medium (Merck, Germany), and allowed to stand overnight. Bacterial growth was confirmed by visual turbidity inspection. Clear bacterial cultures in the nutrient broth became turbid overnight. The tests were done using the original agar-plate test method described by Ames et al. (1975) and included in standard methods for the examination of water and wastewater (APHA, 2005). Results obtained were interpreted based on the established statistical rule that states that “if the number of wells containing bacteria in which the rate of reverse mutation is twice, compared with the mutation rate in the background (or spontaneous) wells, the test sample is considered mutagenic (Ames et al., 1975).” However, in this study, this principle was employed for the classification of mutagenicity based on mutation ratio, which is estimated to be approximately equal to 2, ranging from 1.55 to 1.99, or those that could be rounded off to 0. All experiments were carried out in triplicate. The mutagenicity of the PCs was further classified based on fuzzy logic four-gridded indices (Table 2).

**Table 2:** Classification of mutagenicity, caused by pharmaceutical compounds, based on fuzzy logic principle

Toxic (a)	No mutagenicity (b)	Mutagenic to a lesser extent (c)	Significantly mutagenic (d)
*x<*0.5	*x≥*0.5*≤*1.5	*x*>1.5<2.0	*x≥*2.0

Where $$x \mathrm{\;is\;mutation\;ratio}$$

## Results and discussion

The integrity and healthy functioning of water and soil resources rely on water/soil quality, biotic interaction, habitat structure, energy base, and flow regime, all of which are intertwined. A defect in the integrity and optimum functional performance of environmental resource component affects the entire structure. Of particular interest is understanding the dynamics of how xeno-chemical stressors can affect the chemical quality of natural environments, which may impact habitat structure via biotic interaction and energy flow regimes, thus leading to poor environmental resource quality. Predicting the probable effects of the occurrence of residues of active pharmaceutical compounds in the environment is often elucidated from ecotoxicological bio-assays (Ecotox.) and chemical risk assessment (CRA). Selection of sensitive endpoints to be used for Ecotox. assays and CRA is, therefore, crucial. This is because the selection of endpoints that are insensitive to low environmental concentration levels of chemical stressors, such as APCs, or that are strongly influenced by other co-funding factors within an environmental matrix may be misleading. Thus, the Ecotox. assessment of exposure to lone and combined/potentiated forms of selected APCs, lamivudine, ciprofloxacin, and sulfamethoxazole was conducted using the endpoints *A. cepa*, *D. magna*, and *S. typhimurium* (in the Ames mutagenic test).

## *Allium cepa* (onion) root assay

*Allium cepa* (A. cepa) root is used as a bioindicator for the assessment of potentially genotoxic substances (Nefic et al., 2013; Firbas & Amon, 2014; Fiskesjö, 1988; Leme & Marin-Morales, 2009). Their phytotoxic suitability has been attributed to their relatively large size and appropriateness for the detection of morphological changes (Firbas & Amon, 2014). The root assay was achieved by assessing root lengths (mm) of onions exposed to 10 and 100 µg/L lamivudine, ciprofloxacin, sulfamethoxazole, and LCS-mix test samples, with tap water, used as a control solution.

## Phytotoxic inhibition of root growth of *Allium cepa* bioassay

The test APCs and their mixtures in different combinations were investigated for their phytotoxic characteristics at 10 µg/L and 100 µg/L concentrations, using *A. cepa* bioassays. The root length assay relies on evaluating the root length of *A. cepa* as an indicator endpoint to assess the susceptibility of plants exposed to xenobiotics by evaluating physical impacts on their root growth. The ratio between the root length (mm) (RRL) after and before exposure to test solutions (${~}^{\mathrm{RLAE}}\!\left/ \!{~}_{\mathrm{RLBE}}\right.$) was used as indices for the APCs’ phytotoxicity. Results from the root length assay for 10 µg/L APC test solutions ranged from a ratio of 1.75 ± 0.48 for lamivudine to 2.54 ± 0.97 for sulfamethoxazole, while the same concentration (10 µg/L) of LCS mixture had ratio value of 2.72 ± 1.18 (Table 3). The ratio of the root length (mm) (RRL) of A. cepa after and before exposure to 100 µg/L APCs test solutions ranged from 1.32 ± 0.16 for lamivudine to 2.33 ± 1.01 for ciprofloxacin, while the same 100 µg/L concentration of LCS mixture had RRL value of 2.23 ± 1.10. This suggests that lamivudine had a greater growth rate retarding effect on the root length compared to the other tested solutions, and this increased with concentration.

**Table 3:** *A. cepa* root length before and after exposure to test solutions of selected pharmaceuticals

Test solution	Mean ${~}^{RLAE}\!\left/ \!{~}_{RLBE}\right.$	*P* values relative to control
Control	2.23 ± 0.20	
Lamivudine 10 µg/L	1.75 ± 0.48	0.1818
Lamivudine 100 µg/L	1.32 ± 0.16	0.0036
Ciprofloxacin 10 µg/L	2.03 ± 0.71	0.6569
Ciprofloxacin 100 µg/L	2.33 ± 1.01	0.6866
Sulfamethoxazole 10 µg/L	2.54 ± 0.97	0.6204
Sulfamethoxazole 100 µg/L	2.22 ± 0.70	0.9762
LCS 10 µg/L	2.72 ± 1.18	0.5151
LCS 100 µg/L	2.23 ± 1.10	0.9961

RLAE and RLBE are ratios expressed as root length (mm) after and before exposure, with sample size *n* = 3, respectively

Under controlled conditions, it is expected that the root length should double after 2 days of exposure (ratio of root length, RRL ≥ 2). Analysis of variance (Table 3) indicated that only higher concentration (100 µg/L) of lamivudine exerted a significant negative impact (*p* < 0.05) on the onion root length after exposure, relative to the control in lone PC exposures. This indicates variations in the activities of different pharmaceuticals on the bioindicator *A. cepa*, and the nature of the adverse impact toxicants induced on bioindicators also varies. Hence, there may be a need for a more cut-cross evaluation of toxic ecological impacts of toxicants on a wider flora variety. The impact of the ternary PC combination was not significant (*p* > 0.05). Hence, this APC mixture had no inhibitory effect on the growth of the onion root tip.

## *Allium cepa* root tip microscopic examination

**Fig. 1:**
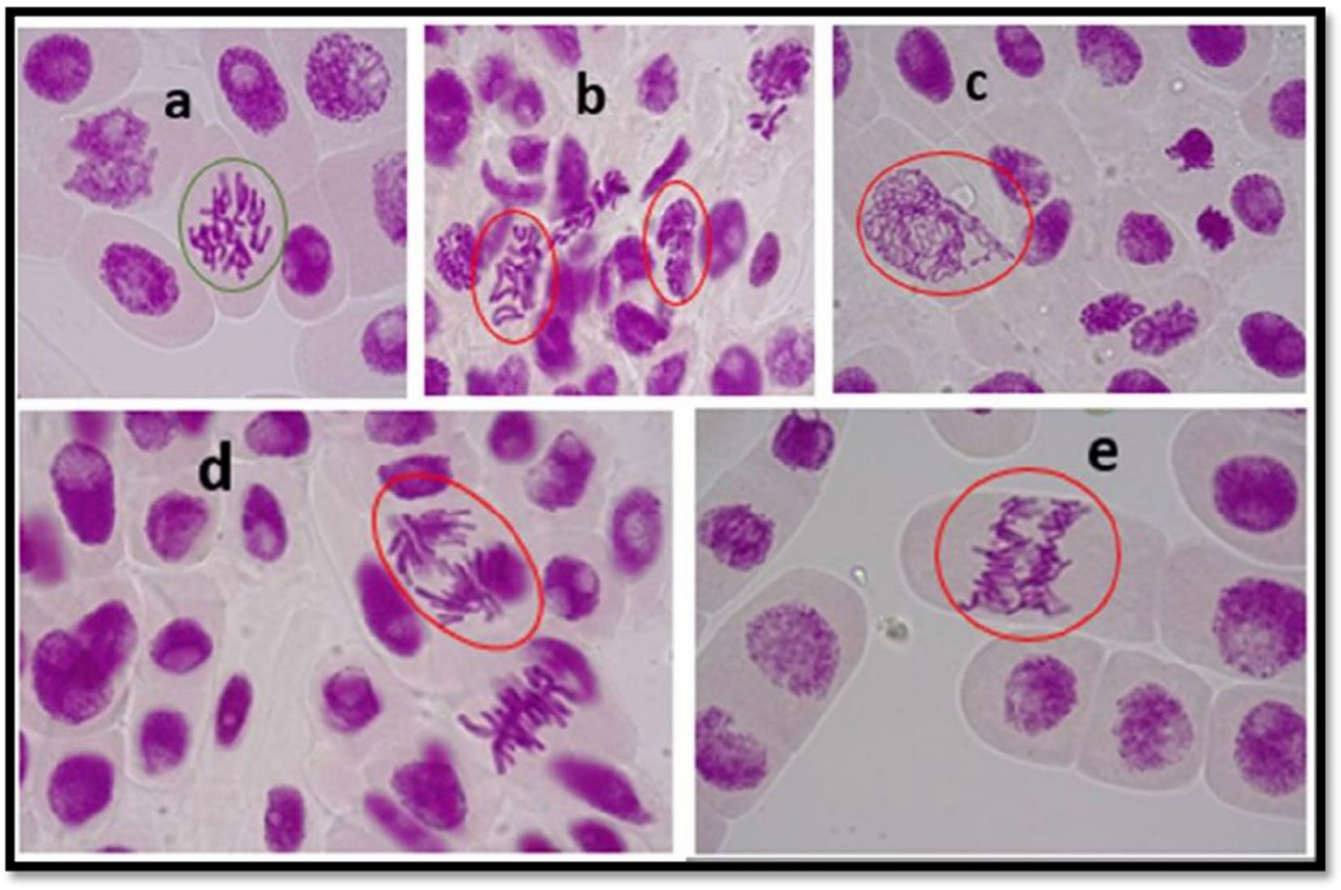
Micrographs of exposed onion cells to lone and mixture PCs: showing chromosomal aberrations in root tip meristem cells of *Allium cepa* exposed to (**a**) tap water, (**b**) 100 µg/L lamivudine, (**c**) 100 µg/L ciprofloxacin, (**d**) 100 µg/L sulfamethoxazole, and (**e**) 100 µg/L LCS, respectively

Microscopic evaluation of *Allium cepa* cells during mitosis was conducted to ascertain the impacts the L, C, and S test samples (lone and mixtures) will have on the genetic materials of the onion roots at cellular levels. These impacts were established by the different chromosomal aberrations induced in the onion cells after the exposure study. The micrograph for the exposed onion cells is presented in Fig. 1.

Normal mitosis phase (anaphase) was observed in the root tip meristem encircled in green (Fig. 1a). However, chromosomal aberrations, including irregular prophase and metaphase; uncoiling chromosome/irregular prophase; vagrant chromosome in anaphase and alignment anaphase; and bridge in anaphase, were observed in the onions root tip meristem exposed to lamivudine, ciprofloxacin, sulfamethoxazole, and LCS mixture, as indicated by the red circles in Fig. 1b, c, d, and e, respectively.

Structural chromosome abnormalities sometimes lead to the loss of genetic materials (Theisen and Shaffer, 2010; Zeiger, 2010; Zeiger, 2018), and this can result in gene rearrangements. These rearrangements may alter the dosage of genes expressed within the affected chromosomal segment, eventually leading to fatal consequences. Study results revealed that the investigated APCs (L, C, S, and LCS mix) were toxic at the molecular (microscopic) levels; however, they were not toxic at the macroscopic level (root length growth), except for lamivudine. Lamivudine showed the ability to induce phytotoxic and genotoxic effects, while ciprofloxacin, sulfamethoxazole, and LCS-mix have the tendencies and capacity to induce genotoxicity only. This indicates that exposure of plants (flora) to residues of pharmaceuticals and other chemical substances could result in genotoxic consequences and not hinder their growth.

## *Daphnia magna* 24–48-h ecological assay (daphnids’ response to individual and mixture PCs)

*Daphnia magna* are very sensitive freshwater crustaceans that are quick to respond to small changes in water chemistry; hence, they are used as endpoints in contaminant exposure studies (Ndlela et al., 2020). Neonates’ of *D. magna* were cultured in specific well plates containing tested individual APCs and their mixtures (4 × 3) at concentrations between 10 and 100 µg/L. A low mean mortality rate of 0.50 ± 0.60 (10%) at 10 µg/L was observed for lamivudine, while the highest mean mortality rate of 5.00 ± 0.00 (100%) at 100 µg/L was observed for lamivudine and the LCS mixture (Table 4).

**Table 4:** Neonates’ mortality of *Daphnia magna* after 24- and 48-h exposure to selected pharmaceuticals

Treatment	Mean mortality (*n* = 20)		Mean % mortality	
	24 h	48 h	24 h	48 h
Control	0.00 ± 0.00	0.00 ± 0.00	0	0
Lamivudine (10 µg/L)	0.50 ± 0.60	2.30 ± 1.00	10	45
Lamivudine (100 µg/L)	4.30 ± 1.00	5.00 ± 0.00	85	100
Ciprofloxacin (10 µg/L)	0.50 ± 0.58	2.75 ± 0.50	10	55
Ciprofloxacin (100 µg/L)	2.5 ± 1.73	3.75 ± 1.26	50	75
Sulfamethoxazole (10 µg/L)	0.75 ± 0.50	1.50 ± 1.73	15	30
Sulfamethoxazole (100 µg/L)	2.50 ± 0.58	4.75 ± 0.50	50	95
LCS (10 µg/L)	0.75 ± 0.50	1.00 ± 0.82	15	20
LCS (100 µg/L)	3.75 ± 0.50	5.00 ± 0.00	75	100

Summarily, the results showed that lamivudine was the most toxic, with 100% toxicity on daphnids’ exposed to 100 µg/L concentrations of the APC solution after 48 h, compared with daphnids’ in the control solution, which remained 100% active (Table 4). Exposure of daphnids’ to 100 µg/L LCS-mix also followed the same fatality trend as that of 100 µg/L lamivudine (Table 4). Comparative exposure data obtained for daphnids’ response to both 10 and 100 µg/L APCs test solutions was fuzzified to normalize imprecisions, uncertainties, and the partial truth associated with the measurements. The basis for the comparison of data from the two test concentrations (10 µg/L and 100 µg/L) in fuzzy logic is on the assumption that the input data are fuzzed by extension principle, optimized, and compared, which allowed for reliable ranking based on the measurement indices (Table 5).

**Table 5:** Classification of lethal impact of targeted compounds using fuzzy logic principle

No lethal impact ($W$)	Mild lethal impact ($X$)	Severe lethal impact ($Y$)	Extremely severe lethal impact ($Z$)
*m*<25	*m* ≥25≤50	*m*>50≤75	*m*>75

Where $$m\;\mathrm{is\;\%\;mortality\;of\;daphnids}$$

The logic pattern of the mortality indices (Table 6) revealed that in all cases, lower concentrations of lamivudine and sulfamethoxazole induced mild (24 h) to severe effects, while no lethal impact, *W* (*m*<25), was observed after 24 h, and a mild lethal impact, *X* (*m*≥25≤50), after 48-h exposure.

**Table 6:** Summary of logic indexed % daphnids response (mortality) grid to each PC

PC solution (10 µg/L)	24-h response	48-h response	PC solution (100 µg/L)	24-h response	48-h response
Lamivudine	W	X	Lamivudine	Z	Z
Ciprofloxacin	W	Y	Ciprofloxacin	X	Y
Sulfamethoxazole	W	X	Sulfamethoxazole	X	Z

Mortality grid indices: *W*, *X*, *Y*, and *Z* mean no lethal impact; mild lethal impact; severe lethal impact and extremely severe lethal impact, respectively.

In contrast, only lamivudine exerted similar fatal effects (extremely severe lethal impact, Z *m*>75) on the daphnids at higher concentrations (100 µg/L) at both 24 h and 48 h. This suggests that lamivudine has the capacity to impact chronic toxicity on the *Daphnia magna* and potentially on aquatic faunas relative to other pharmaceuticals investigated.

**Fig. 2:**
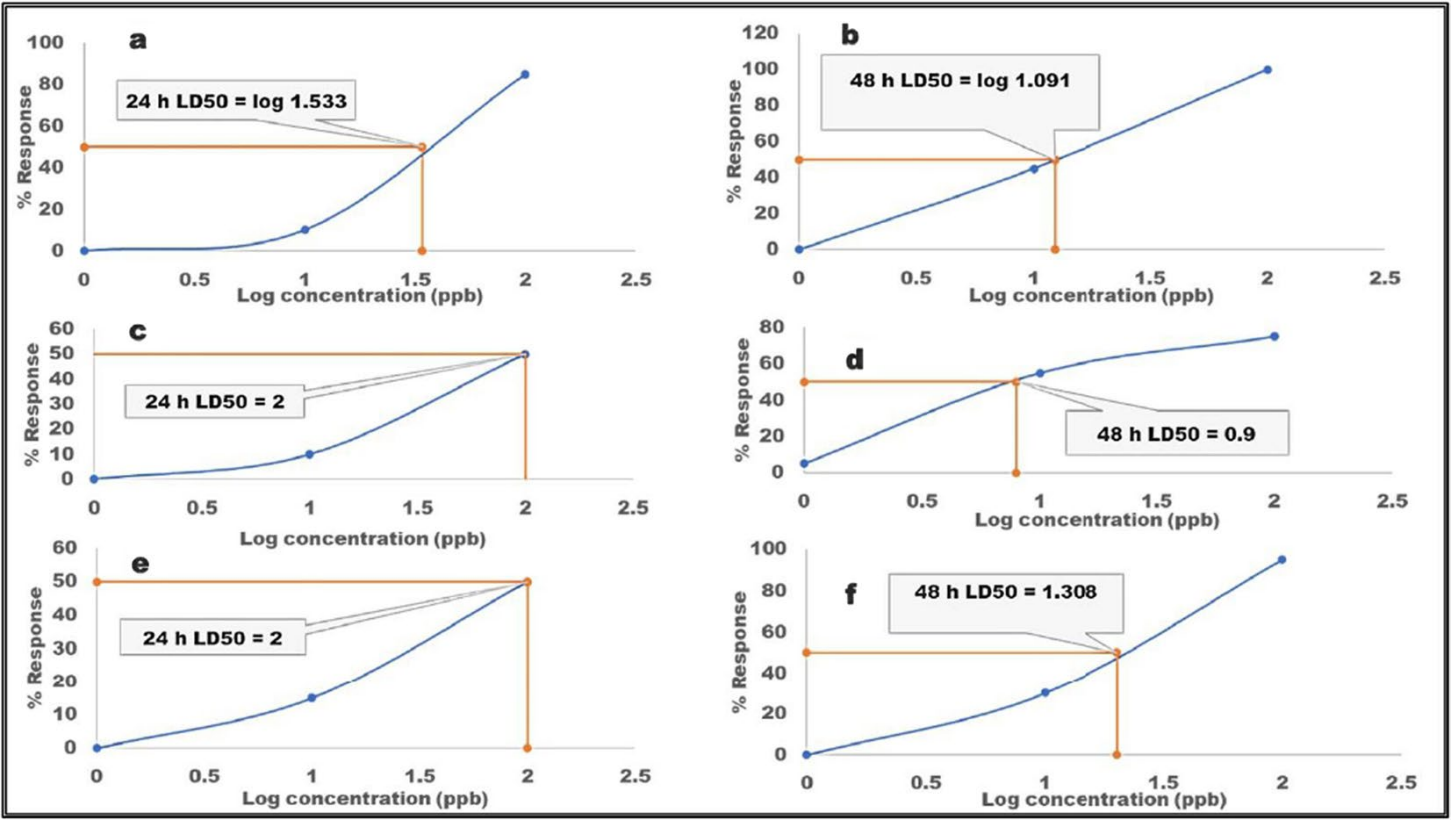
Plot of *Daphnia’s* % response vs log conc. (ppb) to estimate the LD50 (24 and 48 h) curves for (**a**) and (**b**) lamivudine; (**c**) and (**d**) ciprofloxacin; and (**e**) and (**f**) sulfamethoxazole

The ecotoxic attributes of the individual APCs on *Daphnia magna* were investigated by determining the lethal dose (LD50) values. The LD50 values for the APCs were obtained from a plot of the % response (mortality) of daphnids to each APC test solution at 24 and 48 h, against the logarithmic concentration of each test solution. The LD50 curves for individual PCs are presented in Fig. 2.

Ciprofloxacin and sulfamethoxazole induced toxicity on the daphnids at 100 µg/L (log 2) at 24 h, while lamivudine did at 34.1 (log 1.533). The LD50 values at 48 h were 7.94 µg/L (log 0.90) for ciprofloxacin, 12.3 µg/L (log 1.09) for lamivudine, and 20.3 µg/L (log 1.31) for sulfamethoxazole. This revealed lamivudine as most toxic at 24 h and ciprofloxacin fatality at 48 h. The results implied that lamivudine exerts a much more acute effect on the daphnids when compared to ciprofloxacin. Thus, the occurrence of pharmaceuticals, especially lamivudine and ciprofloxacin, at trace levels (in the ppb range) in the fresh aqueous environment has the potential to cause hazardous effects to aquatic biota, including fleas such as *Daphnia*, and the magnitude of negative deleterious effect increases as a function of exposure time.

## Effects of LCS mixture on *Daphnia magna*

The potential of LCS-mix to exert toxic effects on daphnids was also investigated. The data obtained from the *Daphnia magna* bioassays were analyzed and expressed as TUs (toxic units) using the TU model reported by Shakya (2011). The TU concentration values were calculated using the equation; ${\mathrm{TU}}_{\mathrm{A}}= \frac{\mathrm{\%\;A\;in\;the\;mixture\;}\times\;\mathrm{LD}50\mathrm{\;of\;mixture}}{\mathrm{LD}50\mathrm{\;of\;A \;Alone}}\overline{ab}$, where TU_A_ is the toxic unit of substance A in the mixture.

If the sum of the TU of all the constituents in the mixture is equal to 1, the interaction is additive. If the sum of the constituents is less than 1, the interaction is antagonistic, and if the sum is greater than 1, the interaction is synergistic (Shakya, 2011). The combined mixtures tested in this study were of equimolar concentrations (10 or 100 µg/L) of each of the individual PCs. Exposure of daphnids to the LCS-mix followed the same procedure as described for the individual PCs.

**Fig. 3:**
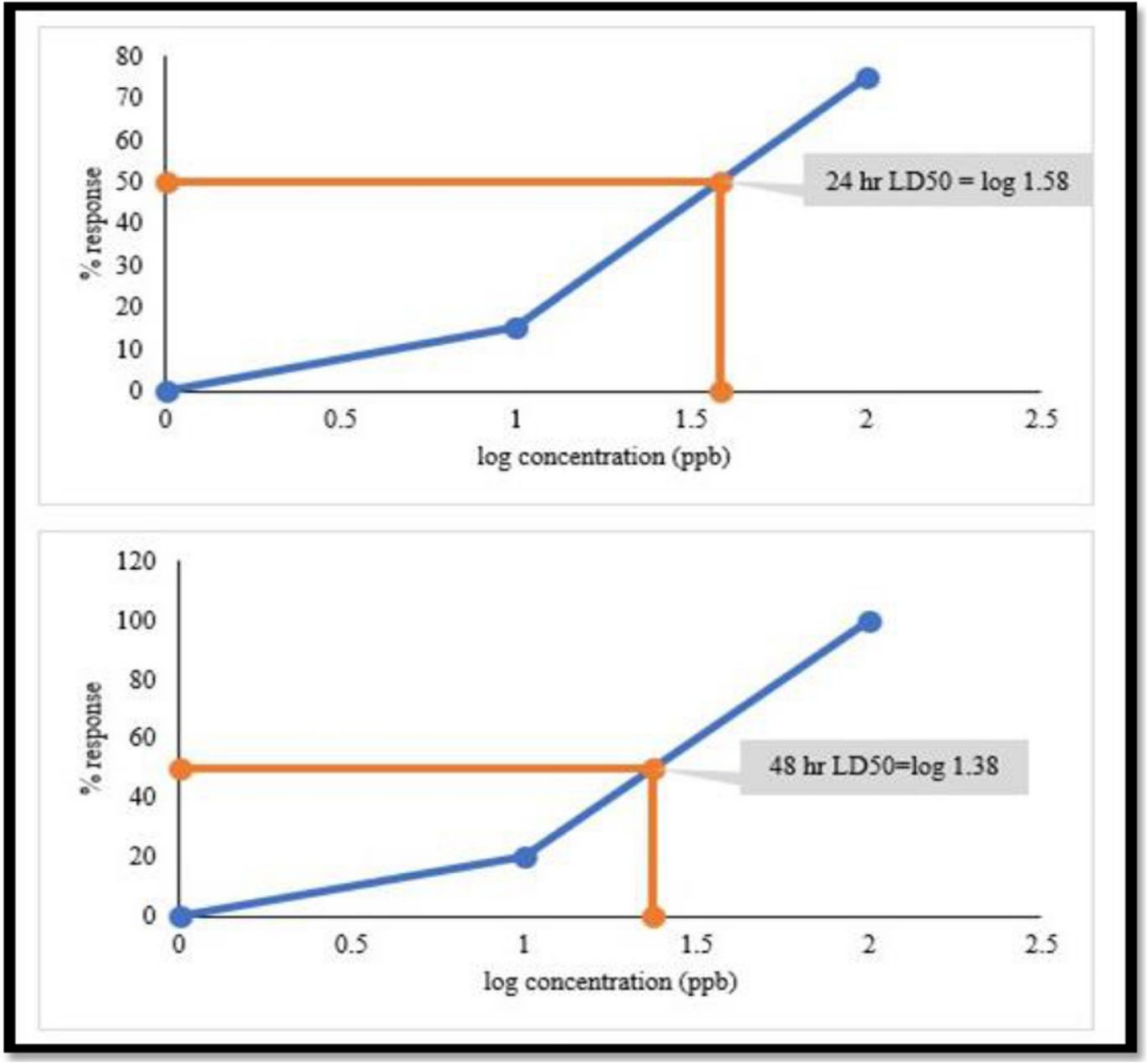
Plot of *Daphnia* response vs log conc (ppb or µg/L) to estimate the 24- and 48-h LD50 of LCS-mixture

The 10 µg/L and 100 µg/L LCS mixtures had a lethal effect on the daphnids. The response pattern is similar to those observed with each of lamivudine, ciprofloxacin, and sulfamethoxazole as depicted by the LD50 curve (Fig. 3, the 24-h LD50 value (log 1.58 = 38 µg/L) showed that the LCS combination mix was more harmful than either of ciprofloxacin or sulfamethoxazole, whose LD50 values were both 100 µg/L at the 24 h).

**Fig. 4:**
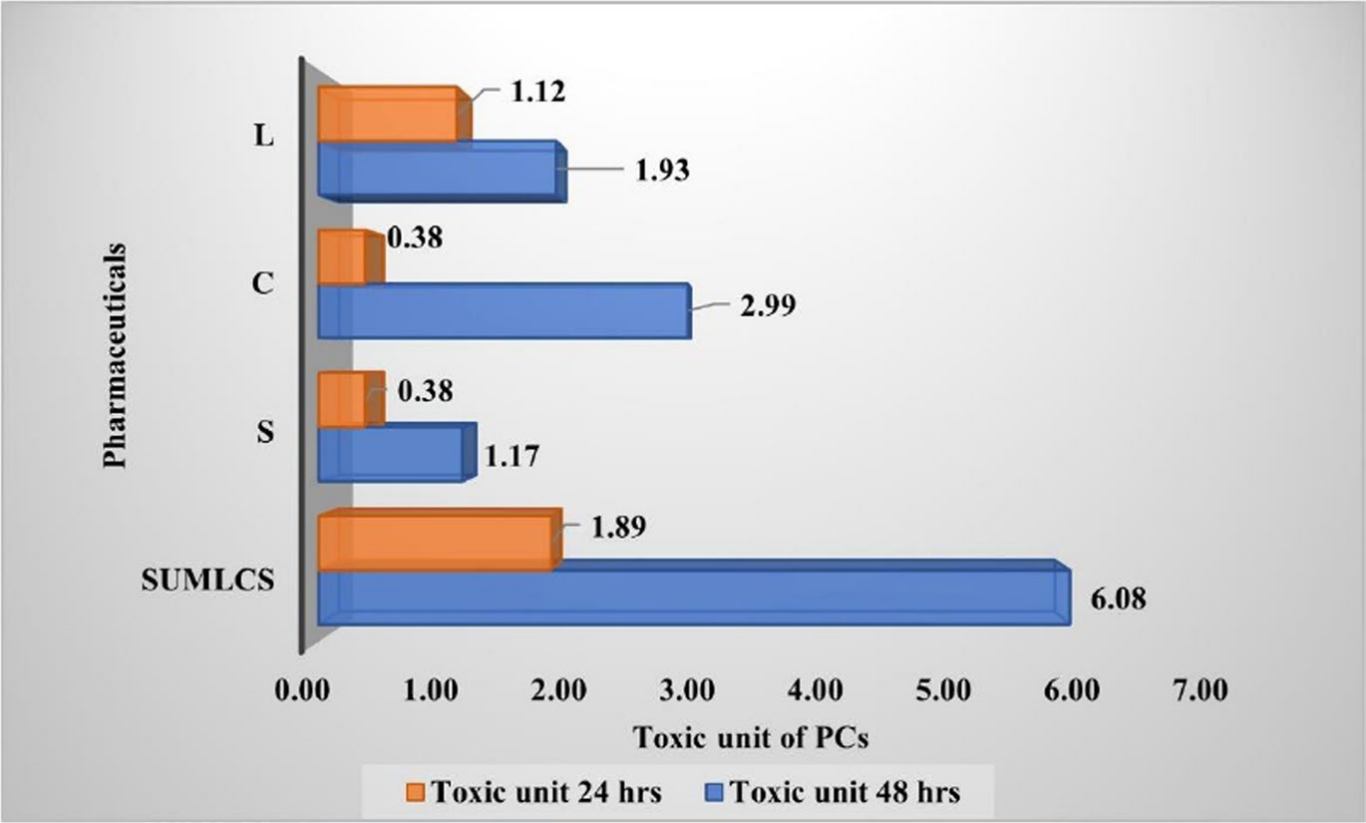
Toxic units of individual and combined pharmaceuticals (LCS)

The calculated sums of the toxic units (TUs) for the 24-h and 48-h daphnid exposure to 10 µg/L and 100 µg/L LCS mixtures were 1.89 and 6.08, respectively (Fig. 4). This toxicity was due to the synergistic effects of the LCS-mix on *D. magna*, since the TU values are greater than 1 (TU > 1).

## Mutagenicity evaluation using Ames *Salmonella* test

Mutagenic substances, often referred to as genotoxins, can cause changes to the DNA structure. Such changes can result in the distortion of transcription and replication of DNA, thus leading to cell death (Basu & Nohmi, 2018). Accordingly, where a substance or compound shows potential for altering the DNA structure of living organisms to inhibit normal functions of such organs, that compound is classified as a mutagen. A phenomenon such as this could eventually lead to the death of the living entity exposed to the mutagens. Hence, the need to evaluate the potentials of LCS and LCS-mix to exert mutagenic toxicity in exposed non-target organisms in the environment.

## Ames *Salmonella* test for lamivudine, ciprofloxacin, and sulfamethoxazole at relevant environmental concentrations using the agar plate method

**Fig. 5:**
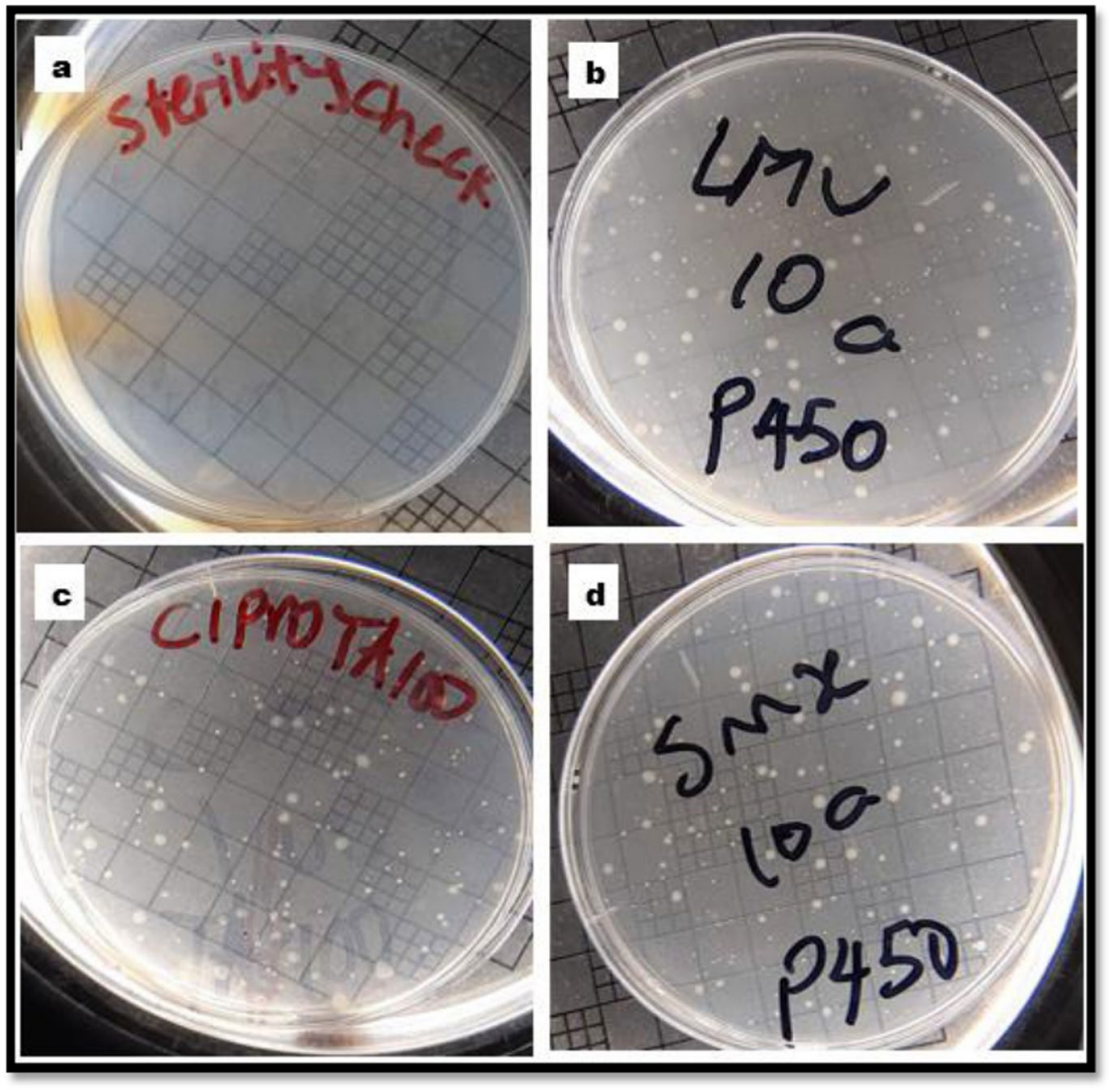
Samples of agar plates on which bacterial colonies were grown: (5a) sterility check or blank (minimal agar + top agar only) with no bacterial colonies; (5b, 5c, and 5d) samples of agar plates with lamivudine, ciprofloxacin, and sulfamethoxazole test solutions, respectively

The mutagenicity of the 3PCs (lamivudine (L), ciprofloxacin (C), and sulfamethoxazole (S)), at concentrations of 10 or 100 µg/L was assessed by exposing three different strains of *Salmonella typhimurium* bacteria, viz., TA 98 P450, TA 100, and TA 100 P450 to each PC for a period of 3 to 5 days using the Ames agar plate method (Fig. 5), which has long been employed in testing frameshift mutagenic toxicity until recently (Ames et al., 1975; Diaz-Baez & Roldan, 1996; Gatehouse, 2012; Resende et al., 2015; Banerjee et al., 2013; Llana-Ruiz-Cabello et al., 2014; Eren and Özata, 2014; Saleem et al., 2015; Levy et al., 2019, Kauffmann et al., 2020). A substance is deemed mutagenic if its mutation rate is twice or more than that of the background.

Results of the exposure of *S. typhimurium* bacterial strains; TA 98P450, TA 100, and TA100 P450 to different concentrations of L, C, and S revealed that the 3PCs were not mutagenic (b, no mutagenicity (ɤ_mr_≥0.5≤1.5)) (at 10 µg/L (Table 7, S1), since the mutation rate of each PC is not as twice more than that of the background.

**Table 7:** Fuzzified (4-grid indices) data from Ames test (agar plate method)

Test solution (10 µg/L)	TA98 P450	TA100	TA100 P450	Test solution (100 µg/L)	TA98 P450	TA100	TA100 P450
Lamivudine	b	b	b	Lamivudine	c	b	b
Ciprofloxacin	b	b	b	Ciprofloxacin	c	b	b
Sulfamethoxazole	b	b	b	Sulfamethoxazole	d	b	b

*a*, cytotoxic (ɤ_mr_ <0.5);* b*, no mutagenicity (ɤ_mr_≥0.5≤1.5) (*c*, mutagenic to a lesser extent (ɤ_mr_>1.5<2.0); (and *d*, significantly mutagenic (ɤ_mr_≥2.0)

Of the three bacterial strains, only the *S. typhimurium* TA98 P450 tested positive with a borderline mutation ratio of 2.0 (d), when exposed to 100 µg/L concentration of sulfamethoxazole, while the same concentrations of lamivudine and ciprofloxacin were to a much lesser extent mutagenic to the TA98 P450 *S. typhimurium* strain (ɤ_mr_>1.5<2.0). However, *S. typhimurium* strains TA 100 and TA100 P450 elicited negative responses from all the tested PCs at 10 and 100 µg/L concentrations. This showed that sulfamethoxazole has the potential to cause an acute frameshift mutation in living organisms, and this could eventually lead to death. In addition, exposure to residues of lamivudine and ciprofloxacin at their environmental occurrence concentrations could cause a chronic frameshift mutation in living organisms, as their mutation ratios were within the range ɤ_mr_ > 1.5 < 2.0 (probable mutagenic potential).

## Effects of PC mixture (LCS) on *Salmonella typhimurium* tester strain

Cocktail mixtures of lamivudine (L), ciprofloxacin (C), and sulfamethoxazole (S) were investigated for their probable mutagenic potential. *Salmonella typhimurium* bacterial strains TA 98 P450, TA 100, or TA100 P450 were exposed to an equimolar concentration (10 and 100 µg/L) ternary cocktail mixtures of the L, C, and S in the Ames test. Data obtained from the responses of the exposed *S. typhimurium* strains to the ternary LCS-mix were interpreted based on the established fuzzified (4-grid) index rule as applicable to the individual PCs.

**Fig. 6:**
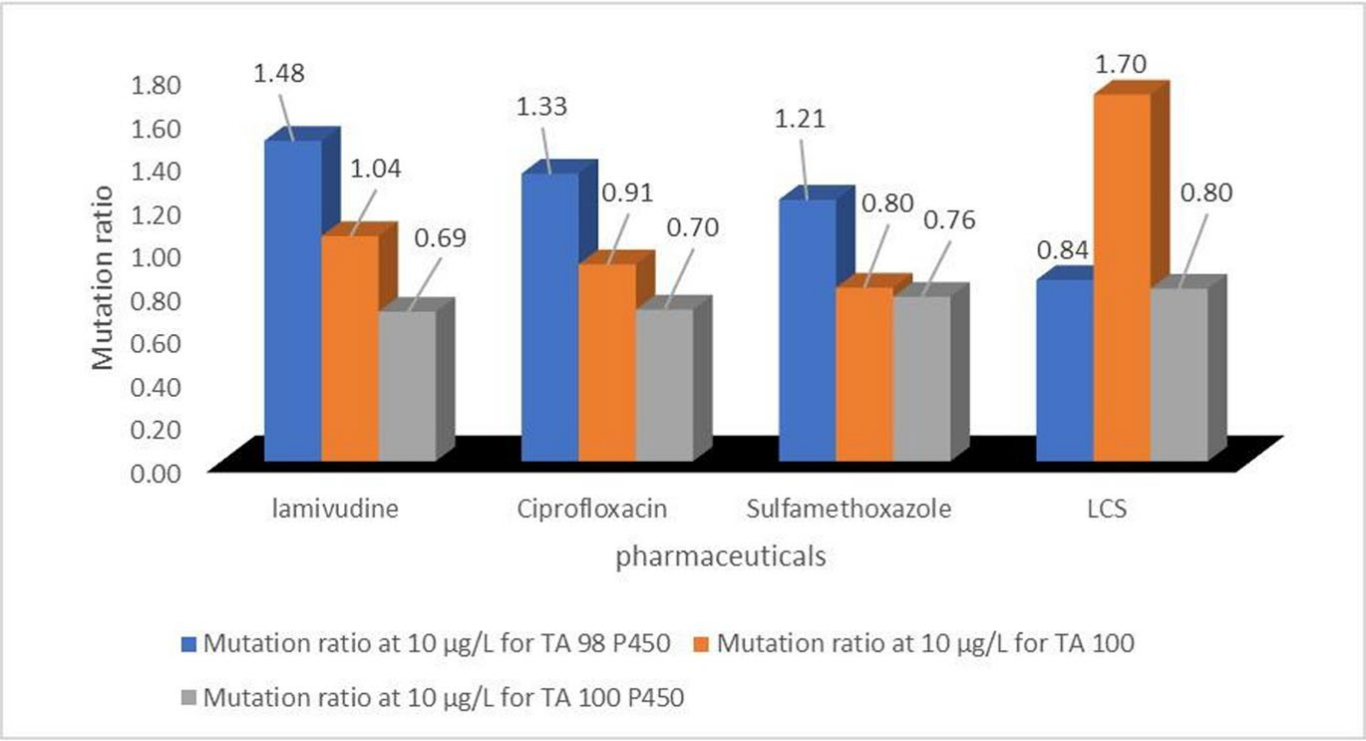
Mutation ratios of 10 µg/L test samples of selected pharmaceuticals

A synergistic toxicity effect was observed in *Salmonella typhimurium* TA100 exposed to 10 µg/L LCS-mix, as evidenced by a mutation ratio of 1.70 (Fig. 6). In contrast, none of the individual PCs had a mutation ratio > 1.5, with respect to other exposed *Salmonella* tester strains. This suggests that extremely low concentrations of the combination of antiretroviral-lamivudine, antibiotics-ciprofloxacin, and sulfamethoxazole could exert a base-pair substitution mutation in living organisms (Yoshida et al., 2016). These findings support earlier observation on the ability of the LCS-mix to exert fatal impact on daphnids at LD50 values of 38 µg/L and 24 µg/L, when exposed for 24 and 48 h, respectively. Therefore, environmental exposure (especially aqueous media) to LCS-mix could be potentially hazardous.

**Fig. 7:**
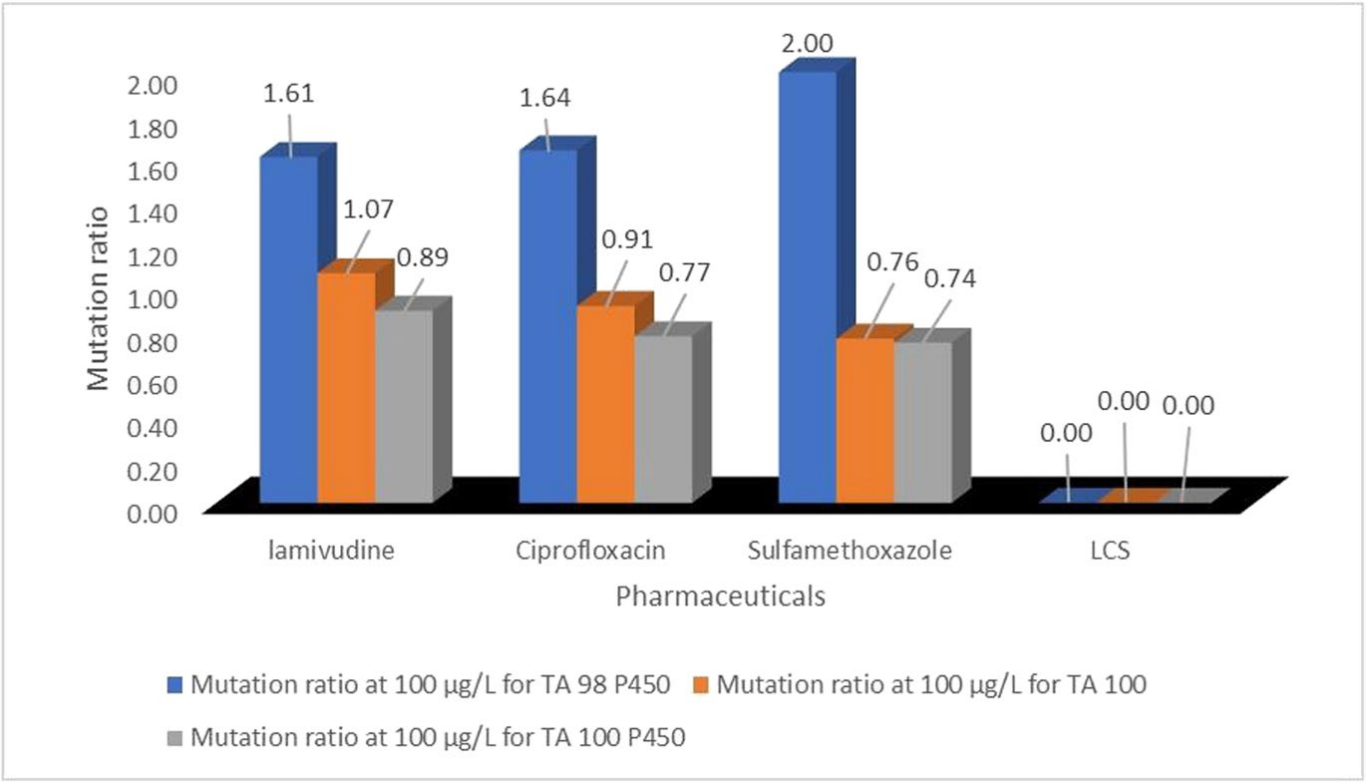
Mutation ratios of 100 µg/L test samples of selected pharmaceuticals

Furthermore, cytotoxicity effects ɤ_mr_>0.5 were observed in all test strains exposed to the 100 µg/L LCS-mix (Fig. 7). This was not unexpected since the LCS-mix contains two antibacterials (ciprofloxacin and sulfamethoxazole). Ordinarily, each of these compounds individually exerts toxic effects on *Daphnia* at 100 µg/L concentration, but their toxic actions were not ascertained at “minute/low concentration (such as 10 µg/L)” to non-therapeutic concentrations. More also, 100 µg/L ciprofloxacin or sulfamethoxazole did not individually exert cytotoxicity on all three tested bacterial strains (TA98 P450, TA100, and TA100 P450), while they did when in combination mixture with lamivudine (Fig. 7). This observation could be accounted for by the presence of lamivudine in the cocktail of LCS-mix, which probably impacted on the mixture some level of toxicity. Since the mechanism of action lamivudine is targeted towards cell replication, it follows that the LCS-mix may be capable of targeting cellular replication by inhibition of DNA synthesis via reverse transcriptase DNA chain termination (Guimarães et al., 2010).

Exposure of the tested three *Salmonella typhimurium* bacterial strains to 10 µg/L of individual lamivudine, ciprofloxacin, and sulfamethoxazole aqueous systems did not exert either mutagenic or cytotoxic effects. The 10 µg/L ternary LCS-mix, however, displayed a mutagenic potential (ɤ_mr_>1.5<2.0) on the TA100 bacterial strain. It was also noted that each of the individual 100 µg/L of lamivudine, ciprofloxacin, and sulfamethoxazole exerted some level of mutagenic activities on only TA98 P450 bacterial strain, whereas their combination (100 µg/L of LCS-mix) exerted a cytotoxic effect on TA98 P450 bacterial strain and the other two bacterial strains (TA100 and TA100 P450). To this end, the ternary combination (LCS-mix) was noted to impact a two-way adverse effect: synergistic effect (mutagenicity) and cytotoxicity, on *Salmonella* test strains, at 10 µg/L and 100 µg/L, respectively.

## Exposure risk evaluation

The exposure risks posed by the tested pharmaceuticals (individually and as a mixture) to the aquatic environment were assessed using different bioassays with three organisms (*A. cepa*, *D. magna*, and three strains of *S. typhimurium*). The assays included growth test and genetic modification, acute toxicity tests, and mutagenicity tests. Results from exposure studies and risk evaluation of lamivudine, ciprofloxacin, sulfamethoxazole, and LCS-mix on the three organisms are summarized (Table 8).

**Table 8:** Summary of bioassays

Samples	*A. cepa* root exposure		Ames test (mutagenicity)			*D. magna*	
	R.L	C.A	TA 98 P450	TA 100	TA 100 P450	% M 24 h	% M 48 h
Lamivudine (10 µg/L)	-	nil	b	b	b	w	x
Lamivudine (100 µg/L)	+	+	c	b	b	z	z
Ciprofloxacin (10 µg/L)	-	-	b	b	b	w	y
Ciprofloxacin (100 µg/L)	-	+	c	b	b	x	y
Sulfamethoxazole (10 µg/L)	-	-	b	b	b	w	x
Sulfamethoxazole (100 µg/L)	-	+	d	b	b	x	z
LCS (10 µg/L)	-	nil	b	c	b	SE	
LCS (100 µg/L)	-	+	a	a	a		

*C.A* means chromosomal aberration; *%M* means percent mortality; (-) indicates no harmful effect; ( +) means fatal impact/chromosomal aberration; *SE* means synergistic effect; *nil* means no experiment conducted; *a*, *b*, *c*, *d*, *w*, *x*, *y*, and *z* are fuzzified results (Tables 2 and 6)

The *A. cepa* root tip assay revealed that all test solutions of LCS and LCS-mix compounds exerted some level of chromosomal aberration, while lamivudine alone had a harmful impact on the root length. *Daphnia magna* toxicity test revealed that severe and extremely severe lethal consequences were exerted by lone pharmaceuticals, while the mixture compound, LCS, impacted a synergistic effect (LCS). The Ames test showed that 100 µg/L sulfamethoxazole was potentially mutagenic to only TA98 P450 among the three *S. typhimurium* strains. Also, both ciprofloxacin and lamivudine were mutagenic to a lesser extent to *S. typhimurium* TA98 P450 bacterial strain, causing frameshift mutation. The combination mixture of LCS had cytotoxic effects on all bacterial strains. This result is not unexpected as both sulfamethoxazole and ciprofloxacin antibiotics are potentially mutagenic in their lone states.

## Correlation among individual (L, C, S) and LCS-mix fatality

**Fig. 8:**
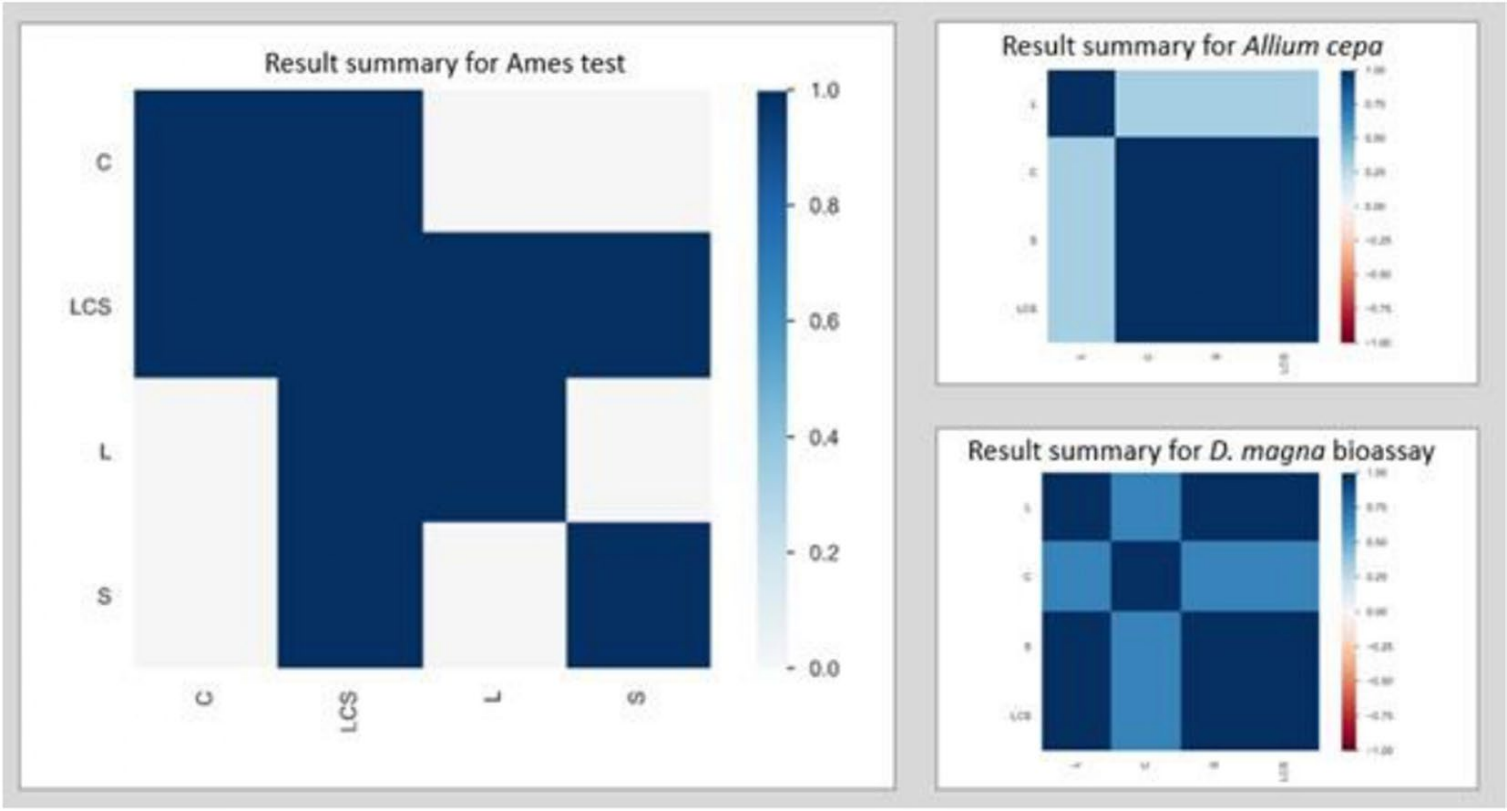
Correlation coefficient measures among individual PCs (L, C, S) and their mixture (LCS)

Kendall’s *T* and Cramér’s *V* correlations were employed to show at a glance which individual PC directly impacts the lethal consequence of LCS mixture for each of the three bioassays. The Kendall rank correlation coefficient (*τ*) measures ordinal association between two variables. Its value lies between − 1 and + 1, − 1 indicating total negative correlation, 0 indicating no correlation, and 1 indicating total positive correlation. Cramér’s *V* correlation is an association measure for nominal random variables. The coefficient ranges from 0 to 1, with 0 indicating independence and 1 indicating perfect association. These correlation methods were employed using Python 3.0 software (Fig. 8).

From the result summary for the Ames test, the correlation measure shows a strong relationship among all the individual PCs and LCS mixture (0.5–0.75), which implies that the cytotoxic effect of LCS-mix was as a result of the combined effects of all three PCs (L, C, and S). A summary of the *A. cepa* test revealed that there was a strong correlation only between ciprofloxacin, sulfamethoxazole, and the LCS-mixture (≤ 0.5), which all had no lethal impact on the growth of onions. However, lamivudine had an obvious fatal phytotoxic impact (> 0.5) on onion growth, howbeit, the impact was annulled by the presence of ciprofloxacin and sulfamethoxazole. Observations from the *D. magna* bioassay indicated that there was a strong correlation (> 0.5) between lamivudine, sulfamethoxazole, and LCS-mix, while ciprofloxacin (< 0.5) had a lesser correlation with the mixture. Thus, the fatal impact caused by exposure of the daphnids to LCS-mix was mainly due to the combination of lamivudine with sulfamethoxazole and ciprofloxacin.

## Conclusion

Toxicity and injury-related responses were observed in *D. magna*, germinating *A. cepa* root tip, mitotic stage *A. cepa*, and *S. typhimurium* bacterial strains. This includes slight irregularities in the root tip growth of *A. cepa*, chromosomal aberration impact on the mitotic phase development of *A. cepa* (onion) root cells, and other genetic impediments, which are all indicative of the potential phyto-toxic and bio-ecological impact that can be induced in exposed plants. The capacity to induce cytotoxicity by the tested PCs (antiretrovirals and antibiotics) in their lone and potentiated forms in some *S. typhimurium* bacterial strains was confirmed in the Ames test. This implies that exposure to lone pharmaceuticals at environmentally relevant concentrations may pose an ecological health risk to both flora and fauna.

In general, the impact of combination of the pharmaceuticals in aqueous ecosystems was greater than when exposed to the tested individual pharmaceutical compounds. The study result showed that these compounds have tendencies to pose a higher risk to exposed living entities when in a combined/potentiated forms, and this could lead to distortion of the regular functioning of the ecosystem, particularly bacterial and other microbial population that are listed among primary producers of the aquatic food web. Plants which constitute a significant group of living entities that maintain and sustain the ecosystem may also be at risk. More also, the potential risk of these contaminants to induce indirect acute or chronic toxic effect on humans and other animals cannot be ruled out or risk exempted, as they form part of the component of the heterotrophic structure and pathways.

Number of revertant colonies (NR) per plate for each *S. typhimurium* bacterial strain.

### Supplementary Information

Below is the link to the electronic supplementary material.

Supplementary file1 (DOCX 16 KB)

## Data Availability

The datasets presented in the current study are available from the corresponding author upon reasonable request.
