# Supplementary material for: Evaluation of the probable synergistic toxicity of selected potentiated antiretroviral and antibiotics on some aquatic biomarker organisms
Source: Environ Monit Assess. 2023 Nov 17;195(12):1489. doi: 10.1007/s10661-023-12068-x (PMC10656311; doi:10.1007/s10661-023-12068-x)
Supplement: Supplementary file 1 — Supplementary file1 (DOCX 16 KB) [file 10661_2023_12068_MOESM1_ESM.docx]

Supplementary information: Number of revertant colonies (NR) per plate for each *S. typhimurium* bacterial strain

| Test samples | TA98 P450 | | | | | | TA 100 | | | | | | | TA 100 P450 | | | | |
| --- | --- | --- | --- | --- | --- | --- | --- | --- | --- | --- | --- | --- | --- | --- | --- | --- | --- | --- |
|  | NR 1 | NR 2 | NR3 | Mean | | S.D | NR 1 | NR 2 | NR3 | Mean | | S.D | | NR 1 | NR 2 | NR3 | Mean | S.D |
| Sterility | 0 | 0 | 0 | 0 | 0 | | 0 | 0 | 0 | 0 | 0 | | 0 | | 0 | 0 | 0 | 0 |
| Background | 9 | 11 | 14 | 11 | 3 | | 130 | 170 | 160 | 153 | 21 | | 340 | | 350 | 320 | 337 | 15 |
| positive control | TNTC | TNTC | TNTC | - | - | | TNTC | TNTC | TNTC | - | - | | TNTC | | TNTC | TNTC | - | - |
| Lamivudine 10 µg/L | 13 | 23 | 13 | 16 | 6 | | 190 | 150 | 140 | 160 | 26 | | 200 | | 200 | 300 | 233 | 58 |
| Lamivudine 100 µg/L | 20 | 15 | 18 | 18 | 3 | | 180 | 150 | 160 | 163 | 15 | | 210 | | 310 | 380 | 300 | 85 |
| Ciprofloxacin 10 µg/L | 8 | 15 | 21 | 15 | 7 | | 140 | 170 | 110 | 140 | 30 | | 240 | | 220 | 250 | 237 | 15 |
| Ciprofloxacin 100 µg/L | 15 | 21 | 18 | 18 | 3 | | 130 | 140 | 150 | 140 | 10 | | 250 | | 270 | 260 | 260 | 10 |
| Sulfamethoxazole 10 µg/L | 21 | 14 | 5 | 13 | 8 | | 100 | 150 | 120 | 123 | 25 | | 270 | | 230 | 270 | 257 | 23 |
| Sulfamethoxazole 100 µg/L | 24 | 20 | 22 | 22 | 2 | | 110 | 110 | 130 | 117 | 12 | | 310 | | 220 | 220 | 250 | 52 |
| LCS 10 µg/L | 0 | 0 | 0 | 0 | 0 | | 0 | 0 | 1 | 0 | 1 | | 0 | | 0 | 0 | 0 | 0 |
| LCS 100 µg/L | 11 | 12 | 13 | 12 | 1 | | 250 | 260 | 290 | 267 | 21 | | 330 | | 330 | 210 | 290 | 69 |

S.D means standard deviation; TNTC –Too numerous to count
